# (*E*)-1-Benzyl­idene-2,2-diphenyl­hydrazine

**DOI:** 10.1107/S1600536812000657

**Published:** 2012-01-18

**Authors:** Angel Mendoza, Ruth Meléndrez-Luevano, Blanca M. Cabrera-Vivas, Claudia D. Lozano-Márquez, Vladimir Carranza

**Affiliations:** a Centro de Química, Instituto de Ciencias, Benemérita Universidad Autónoma de Puebla, Puebla, Pue, México; b Facultad de Ciencias Químicas, Benemérita Universidad Autónoma de Puebla, Puebla, Pue, México

## Abstract

The asymmetric unit of the title compound, C_19_H_16_N_2_, contains two independent mol­ecules, both of which show an *E* configuration with respect to the C=N bond. The dihedral angles between the phenyl rings bonded to the hydrazine group are 81.00 (10) and 88.34 (8)° in the two mol­ecules. Inter­molecular C—H⋯π inter­actions are observed in the crystal structure.

## Related literature

For biological applications of hydrazones, see: Guniz & Rollas (2002 ▶); Ibañez *et al.* (2002 ▶); Vicini *et al.* (2002 ▶); Rollas *et al.* (2002 ▶). For related structures, see: Clulow *et al.* (2008 ▶); Mendoza *et al.* (2011 ▶). For bond-length data, see: Allen *et al.* (1987 ▶).
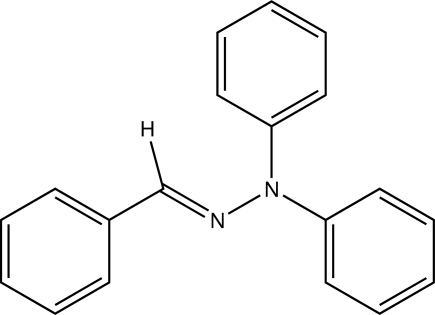


## Experimental

### 

#### Crystal data

C_19_H_16_N_2_
*M* *_r_* = 272.34
Triclinic, 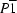

*a* = 10.283 (3) Å
*b* = 10.558 (3) Å
*c* = 16.409 (5) Å
α = 75.70 (4)°
β = 85.40 (2)°
γ = 63.403 (15)°
*V* = 1542.6 (8) Å^3^
*Z* = 4
Mo *K*α radiation
μ = 0.07 mm^−1^
*T* = 298 K
0.5 × 0.4 × 0.2 mm

#### Data collection

Siemens P4 diffractometer
Absorption correction: ψ scan (North *et al.*, 1968 ▶) *T*_min_ = 0.924, *T*_max_ = 0.97
9357 measured reflections
8060 independent reflections
4867 reflections with *I* > 2σ(*I*)
*R*_int_ = 0.089
3 standard reflections every 97 reflections intensity decay: 1%

#### Refinement

*R*[*F* ^2^ > 2σ(*F* ^2^)] = 0.056
*wR*(*F* ^2^) = 0.176
*S* = 1.02
8060 reflections
380 parameters
H-atom parameters constrained
Δρ_max_ = 0.16 e Å^−3^
Δρ_min_ = −0.21 e Å^−3^

### 

Data collection: *XSCANS* (Siemens, 1994 ▶); cell refinement: *XSCANS*; data reduction: *XSCANS*; program(s) used to solve structure: *SIR2004* (Burla *et al.*, 2005 ▶); program(s) used to refine structure: *SHELXL97* (Sheldrick, 2008 ▶); molecular graphics: *ORTEP-3* (Farrugia, 1997 ▶); software used to prepare material for publication: *WinGX* (Farrugia, 1999 ▶).

## Supplementary Material

Crystal structure: contains datablock(s) global, I. DOI: 10.1107/S1600536812000657/is5046sup1.cif

Structure factors: contains datablock(s) I. DOI: 10.1107/S1600536812000657/is5046Isup2.hkl

Supplementary material file. DOI: 10.1107/S1600536812000657/is5046Isup3.cml

Additional supplementary materials:  crystallographic information; 3D view; checkCIF report

## Figures and Tables

**Table 1:** Hydrogen-bond geometry (Å, °) *Cg*1, *Cg*2 and *Cg*4 are the centroids of the C1–C6, C7–C12 and C20–C25 rings, respectively.

*D*—H⋯*A*	*D*—H	H⋯*A*	*D*⋯*A*	*D*—H⋯*A*
C15—H15⋯*Cg*4^i^	0.93	2.93	3.851 (2)	171
C22—H22⋯*Cg*2^ii^	0.93	2.88	3.788 (3)	166
C37—H37⋯*Cg*1^iii^	0.93	2.99	3.828 (2)	150

Symmetry codes: (i) 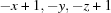; (ii) 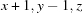; (iii) 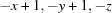.

**Fig. 1.**
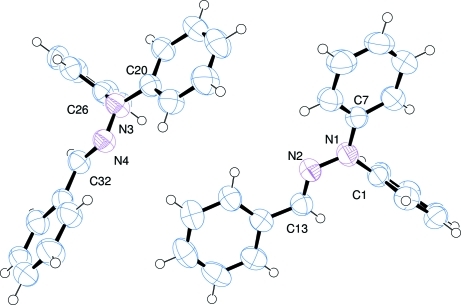
The molecular structure of title compound, with atom labels and 50% probability displacement ellipsoids for non-H atoms.

## supplementary crystallographic information

### Comment

Different applications of hydrazones has been demonstrated in the pharmaceutical and microbiological industry (Guniz *et al.*, 2002; Ibañez *et al.*, 2002). The structure of hydrazones is directly related to their activity. The condensation reaction with aromatic aldehydes in order to produce hydrazones has antibacterial and antifungal activity. Antimicrobial activity is enhanced when aldehydes have functional groups like –NO_2_ and –Cl (Vicini *et al.*, 2002; Rollas *et al.*, 2002).

The asymmetric unit of the title compound contains two non-planar molecules. Each molecule shows an *E* configuration on the C═N double bond. The dihedral angle between the phenyl rings, C1–C6 and C7–C12, is 81.00 (10)° for molecule 1, and that between C20–C25 and C26–C31 rings is 88.34 (8)° for molecule 2. The phenyl rings attached to imine group shows a little twist with respect to the C═N bond, with torsion angles of 5.7 (2)° for N4—C32—C33—C34 and 5.9 (2)° for N2—C13—C14—C19. The N—N distances [N1—N2 1.3689 (18) Å molecule 1 and N3—N4 1.3681 (18) Å molecule 2] are shorter than found in free diphenylhydrazine [1.418 (2) Å] (Clulow *et al.*, 2008). Imine bond distances [N2—C13 1.279 (2) Å for molecule 1 and N4—C32 1.278 (2) Å for molecule 2] are longer than N=C typical bond (Allen *et al.*, 1987) but similar to the structure with *N*,*N*-diphenylhydrazone group reported previously (Mendoza *et al.*, 2011). Intermolecular C—H···π interactions are also observed.

### Experimental

Diphenylhydrazine was dissolved in ethanol (1.2 chemical equivalents). A chemical equivalent of aldehyde, previously dissolved in the same solvent, was added drop by drop with continuous stirring. The reaction mixture was kept at room temperature and was monitored by TLC. After three hours the amber solution turns to be precipitated. The mixture was separated by filtration in a vacuum system and the precipitate was washed three times with cold methanol. The hydrazones were recrystallized with acetonitrile by a continuous and controlled process until colorless crystals with adequate size were developed in order to obtain X-ray studies. Yield 80%.

### Refinement

H atoms were placed in geometrically idealized positions and refined as riding on their parent atoms, with C—H = 0.93 Å, and with *U*_iso_(H) = 1.2*U*_eq_(C).

### Crystal data

**Table:**

C_19_H_16_N_2_	*Z* = 4
*M**_r_* = 272.34	*F*(000) = 576
Triclinic, *P*1	*D*_x_ = 1.173 Mg m^−^^3^
*a* = 10.283 (3) Å	Mo *K*α radiation, λ = 0.71073 Å
*b* = 10.558 (3) Å	Cell parameters from 41 reflections
*c* = 16.409 (5) Å	θ = 4.3–12.6°
α = 75.70 (4)°	µ = 0.07 mm^−^^1^
β = 85.40 (2)°	*T* = 298 K
γ = 63.403 (15)°	Prism, colorless
*V* = 1542.6 (8) Å^3^	0.5 × 0.4 × 0.2 mm

### Data collection

**Table:**

Siemens P4 diffractometer	*R*_int_ = 0.089
graphite	θ_max_ = 29.0°, θ_min_ = 2.2°
2θ/ω scans	*h* = −1→13
Absorption correction: ψ scan (North *et al.*, 1968)	*k* = −12→13
*T*_min_ = 0.924, *T*_max_ = 0.97	*l* = −22→22
9357 measured reflections	3 standard reflections every 97 reflections
8060 independent reflections	intensity decay: 1%
4867 reflections with *I* > 2σ(*I*)	

### Refinement

**Table:**

Refinement on *F*^2^	Secondary atom site location: difference Fourier map
Least-squares matrix: full	Hydrogen site location: inferred from neighbouring sites
*R*[*F*^2^ > 2σ(*F*^2^)] = 0.056	H-atom parameters constrained
*wR*(*F*^2^) = 0.176	*w* = 1/[σ^2^(*F*_o_^2^) + (0.0803*P*)^2^ + 0.1084*P*] where *P* = (*F*_o_^2^ + 2*F*_c_^2^)/3
*S* = 1.02	(Δ/σ)_max_ < 0.001
8060 reflections	Δρ_max_ = 0.16 e Å^−^^3^
380 parameters	Δρ_min_ = −0.21 e Å^−^^3^
0 restraints	Extinction correction: *SHELXL*
Primary atom site location: structure-invariant direct methods	Extinction coefficient: 0.019 (3)

### Special details

**Table:**

Experimental. UV λ_max_ = 340.13 nm. FT IR (film): (cm^-1^): 1586, 1490 n(C=N). ^1^H NMR (400 MHz, (CD_3_)_2_CO: (d/p.p.m.): 7.64–7.62 (m, 2H), 7.48–7.44 (m, 4H), 7.36–7.33 (m, 2H), 7.28–7.18 (m, 8H). ^13^C NMR (400 MHz, (CD_3_)_2_CO: (d/ p.p.m.): 143.66, 136.26, 135.18, 129.87, 128.55, 128.11, 126.18, 124.58, 122.35. MS—EI: m/*z* = 272 *M*^+^. C_19_H_16_N_2_.
Geometry. All s.u.'s (except the s.u. in the dihedral angle between two l.s. planes) are estimated using the full covariance matrix. The cell s.u.'s are taken into account individually in the estimation of s.u.'s in distances, angles and torsion angles; correlations between s.u.'s in cell parameters are only used when they are defined by crystal symmetry. An approximate (isotropic) treatment of cell s.u.'s is used for estimating s.u.'s involving l.s. planes.
Refinement. Refinement of *F*^2^ against ALL reflections. The weighted *R*-factor *wR* and goodness of fit *S* are based on *F*^2^, conventional *R*-factors *R* are based on *F*, with *F* set to zero for negative *F*^2^. The threshold expression of *F*^2^ > 2σ(*F*^2^) is used only for calculating *R*-factors(gt) *etc*. and is not relevant to the choice of reflections for refinement. *R*-factors based on *F*^2^ are statistically about twice as large as those based on *F*, and *R*- factors based on ALL data will be even larger.

### Fractional atomic coordinates and isotropic or equivalent isotropic displacement parameters (Å^2^)

**Table:**

	*x*	*y*	*z*	*U*_iso_*/*U*_eq_	
N4	0.66701 (13)	0.13335 (14)	0.09112 (8)	0.0577 (3)	
C20	0.63112 (16)	0.02550 (15)	0.23094 (9)	0.0553 (3)	
C14	0.75034 (16)	0.49413 (16)	0.28985 (10)	0.0599 (4)	
C33	0.71087 (16)	0.25152 (16)	−0.04454 (9)	0.0555 (3)	
N3	0.57712 (13)	0.09670 (16)	0.14774 (8)	0.0648 (3)	
N1	0.74949 (16)	0.36007 (14)	0.51727 (8)	0.0670 (4)	
N2	0.75818 (14)	0.36776 (13)	0.43264 (8)	0.0593 (3)	
C32	0.61652 (16)	0.21588 (16)	0.01844 (9)	0.0579 (4)	
H32	0.5178	0.2539	0.0055	0.069*	
C7	0.78137 (16)	0.22139 (17)	0.57040 (10)	0.0582 (4)	
C13	0.74545 (17)	0.48540 (17)	0.38070 (10)	0.0617 (4)	
H13	0.7331	0.5653	0.4004	0.074*	
C1	0.73689 (17)	0.47714 (16)	0.55251 (9)	0.0575 (4)	
C38	0.65030 (19)	0.34881 (17)	−0.12140 (10)	0.0653 (4)	
H38	0.5501	0.3916	−0.1313	0.078*	
C25	0.54528 (17)	−0.01815 (17)	0.28984 (10)	0.0636 (4)	
H25	0.4526	0	0.2739	0.076*	
C3	0.8446 (2)	0.5908 (2)	0.61514 (11)	0.0714 (4)	
H3	0.927	0.5924	0.6335	0.086*	
C31	0.32350 (18)	0.27985 (18)	0.13879 (11)	0.0672 (4)	
H31	0.3513	0.3366	0.1618	0.081*	
C27	0.38484 (17)	0.06357 (17)	0.09176 (10)	0.0609 (4)	
H27	0.4541	−0.0258	0.0833	0.073*	
C15	0.75028 (19)	0.61704 (19)	0.23438 (11)	0.0735 (5)	
H15	0.746	0.6933	0.255	0.088*	
C37	0.7370 (2)	0.38223 (18)	−0.18280 (10)	0.0704 (4)	
H37	0.6947	0.449	−0.2332	0.084*	
C6	0.60157 (18)	0.58601 (19)	0.56083 (11)	0.0682 (4)	
H6	0.5189	0.5855	0.542	0.082*	
C5	0.58795 (19)	0.69641 (19)	0.59711 (12)	0.0734 (5)	
H5	0.4962	0.7695	0.603	0.088*	
C24	0.5964 (2)	−0.08800 (19)	0.37158 (11)	0.0759 (5)	
H24	0.5381	−0.117	0.4103	0.091*	
C19	0.75539 (19)	0.38216 (19)	0.25709 (11)	0.0695 (4)	
H19	0.7548	0.2993	0.2932	0.083*	
C34	0.86116 (18)	0.1890 (2)	−0.03245 (11)	0.0704 (4)	
H34	0.9044	0.125	0.0185	0.085*	
C2	0.85940 (18)	0.48028 (18)	0.57871 (10)	0.0652 (4)	
H2	0.9514	0.4085	0.5719	0.078*	
C16	0.7566 (2)	0.6266 (2)	0.14858 (12)	0.0849 (6)	
H16	0.757	0.7091	0.1119	0.102*	
C36	0.8848 (2)	0.3178 (2)	−0.17017 (11)	0.0780 (5)	
H36	0.9432	0.3393	−0.2121	0.094*	
C21	0.76949 (18)	−0.00257 (19)	0.25626 (11)	0.0687 (4)	
H21	0.8288	0.0257	0.218	0.082*	
C26	0.42597 (15)	0.14762 (16)	0.12623 (9)	0.0544 (3)	
C12	0.8288 (2)	0.09859 (18)	0.53921 (11)	0.0715 (4)	
H12	0.8359	0.1065	0.4815	0.086*	
C17	0.76235 (19)	0.5146 (2)	0.11706 (12)	0.0821 (6)	
H17	0.7669	0.5212	0.0594	0.099*	
C8	0.7669 (2)	0.2061 (2)	0.65620 (11)	0.0803 (5)	
H8	0.7307	0.2881	0.6783	0.096*	
C4	0.7097 (2)	0.69792 (19)	0.62438 (11)	0.0704 (4)	
H4	0.7007	0.7717	0.6491	0.084*	
C29	0.13758 (18)	0.2447 (2)	0.08227 (11)	0.0707 (4)	
H29	0.0405	0.2776	0.0672	0.085*	
C28	0.24001 (18)	0.11309 (19)	0.06995 (11)	0.0687 (4)	
H28	0.2121	0.0566	0.0468	0.082*	
C30	0.17860 (18)	0.32819 (19)	0.11703 (12)	0.0748 (5)	
H30	0.1089	0.4172	0.1259	0.09*	
C18	0.7613 (2)	0.3935 (2)	0.17156 (12)	0.0809 (5)	
H18	0.7646	0.318	0.1505	0.097*	
C35	0.9464 (2)	0.2210 (2)	−0.09511 (12)	0.0847 (6)	
H35	1.0469	0.1766	−0.0866	0.102*	
C9	0.8057 (3)	0.0704 (2)	0.70945 (12)	0.0894 (6)	
H9	0.7979	0.0617	0.7672	0.107*	
C11	0.8656 (2)	−0.0360 (2)	0.59365 (14)	0.0867 (6)	
H11	0.8983	−0.1181	0.5719	0.104*	
C10	0.8554 (2)	−0.0514 (2)	0.67862 (13)	0.0843 (6)	
H10	0.8816	−0.1428	0.7147	0.101*	
C22	0.8179 (2)	−0.0727 (2)	0.33864 (13)	0.0862 (6)	
H22	0.9104	−0.0914	0.3552	0.103*	
C23	0.7331 (2)	−0.1152 (2)	0.39642 (12)	0.0864 (6)	
H23	0.7672	−0.162	0.4517	0.104*	

### Atomic displacement parameters (Å^2^)

**Table:**

	*U*^11^	*U*^22^	*U*^33^	*U*^12^	*U*^13^	*U*^23^
N4	0.0519 (7)	0.0667 (7)	0.0524 (7)	−0.0265 (6)	0.0002 (5)	−0.0097 (6)
C20	0.0544 (8)	0.0515 (8)	0.0517 (8)	−0.0179 (6)	−0.0030 (6)	−0.0077 (6)
C14	0.0514 (8)	0.0574 (8)	0.0584 (9)	−0.0166 (7)	0.0011 (6)	−0.0067 (7)
C33	0.0595 (9)	0.0565 (8)	0.0510 (8)	−0.0257 (7)	−0.0009 (6)	−0.0126 (6)
N3	0.0496 (7)	0.0842 (9)	0.0530 (7)	−0.0297 (6)	−0.0034 (5)	−0.0017 (6)
N1	0.0903 (10)	0.0572 (7)	0.0514 (7)	−0.0310 (7)	−0.0002 (6)	−0.0116 (6)
N2	0.0620 (7)	0.0589 (7)	0.0521 (7)	−0.0234 (6)	0.0010 (5)	−0.0113 (6)
C32	0.0521 (8)	0.0631 (9)	0.0534 (8)	−0.0224 (7)	−0.0034 (6)	−0.0095 (7)
C7	0.0593 (8)	0.0601 (9)	0.0571 (8)	−0.0301 (7)	−0.0006 (6)	−0.0091 (7)
C13	0.0643 (9)	0.0572 (9)	0.0596 (9)	−0.0240 (7)	0.0009 (7)	−0.0124 (7)
C1	0.0635 (9)	0.0561 (8)	0.0517 (8)	−0.0261 (7)	0.0016 (6)	−0.0115 (6)
C38	0.0677 (10)	0.0621 (9)	0.0566 (9)	−0.0222 (8)	−0.0035 (7)	−0.0090 (7)
C25	0.0591 (9)	0.0626 (9)	0.0599 (9)	−0.0218 (7)	−0.0001 (7)	−0.0084 (7)
C3	0.0724 (11)	0.0759 (11)	0.0749 (11)	−0.0423 (9)	−0.0052 (8)	−0.0122 (9)
C31	0.0669 (10)	0.0660 (10)	0.0701 (10)	−0.0274 (8)	−0.0015 (8)	−0.0211 (8)
C27	0.0580 (9)	0.0611 (9)	0.0576 (9)	−0.0205 (7)	0.0024 (7)	−0.0153 (7)
C15	0.0702 (10)	0.0670 (10)	0.0708 (11)	−0.0260 (8)	0.0032 (8)	−0.0037 (8)
C37	0.0917 (13)	0.0649 (10)	0.0504 (9)	−0.0339 (9)	0.0007 (8)	−0.0076 (7)
C6	0.0591 (9)	0.0722 (10)	0.0738 (10)	−0.0273 (8)	−0.0021 (8)	−0.0203 (8)
C5	0.0656 (10)	0.0679 (10)	0.0817 (12)	−0.0226 (8)	0.0110 (8)	−0.0257 (9)
C24	0.0823 (12)	0.0702 (10)	0.0585 (10)	−0.0261 (9)	0.0047 (8)	−0.0026 (8)
C19	0.0743 (11)	0.0676 (10)	0.0586 (9)	−0.0260 (8)	−0.0003 (8)	−0.0112 (8)
C34	0.0635 (10)	0.0920 (12)	0.0564 (9)	−0.0396 (9)	−0.0037 (7)	−0.0069 (8)
C2	0.0569 (9)	0.0624 (9)	0.0690 (10)	−0.0230 (7)	0.0023 (7)	−0.0100 (7)
C16	0.0705 (11)	0.0873 (13)	0.0689 (11)	−0.0267 (10)	0.0016 (9)	0.0129 (10)
C36	0.0918 (13)	0.1001 (13)	0.0590 (10)	−0.0592 (11)	0.0123 (9)	−0.0182 (9)
C21	0.0595 (9)	0.0724 (10)	0.0663 (10)	−0.0281 (8)	−0.0115 (7)	−0.0008 (8)
C26	0.0475 (7)	0.0624 (8)	0.0484 (7)	−0.0225 (6)	0.0000 (6)	−0.0077 (6)
C12	0.0849 (12)	0.0635 (10)	0.0661 (10)	−0.0344 (9)	0.0062 (8)	−0.0133 (8)
C17	0.0622 (10)	0.1023 (15)	0.0548 (10)	−0.0176 (10)	−0.0015 (7)	−0.0085 (10)
C8	0.1113 (15)	0.0789 (12)	0.0610 (10)	−0.0526 (11)	0.0066 (9)	−0.0146 (9)
C4	0.0890 (12)	0.0679 (10)	0.0635 (10)	−0.0412 (9)	0.0080 (8)	−0.0203 (8)
C29	0.0521 (9)	0.0836 (11)	0.0682 (10)	−0.0246 (8)	−0.0039 (7)	−0.0132 (9)
C28	0.0672 (10)	0.0787 (11)	0.0669 (10)	−0.0358 (9)	−0.0051 (8)	−0.0193 (8)
C30	0.0588 (10)	0.0677 (10)	0.0801 (12)	−0.0104 (8)	0.0022 (8)	−0.0215 (9)
C18	0.0795 (12)	0.0866 (12)	0.0641 (11)	−0.0241 (10)	−0.0019 (9)	−0.0201 (9)
C35	0.0685 (11)	0.1229 (16)	0.0691 (11)	−0.0526 (11)	0.0020 (9)	−0.0135 (11)
C9	0.1234 (17)	0.1000 (15)	0.0604 (10)	−0.0717 (14)	−0.0005 (10)	−0.0010 (10)
C11	0.1050 (15)	0.0634 (11)	0.0913 (15)	−0.0409 (10)	0.0075 (11)	−0.0116 (10)
C10	0.0920 (13)	0.0766 (12)	0.0842 (13)	−0.0506 (11)	−0.0073 (10)	0.0103 (10)
C22	0.0771 (12)	0.0893 (13)	0.0755 (12)	−0.0306 (10)	−0.0280 (10)	0.0063 (10)
C23	0.0938 (14)	0.0823 (12)	0.0595 (10)	−0.0260 (11)	−0.0161 (10)	0.0041 (9)

### Geometric parameters (Å, °)

**Table:**

N4—C32	1.278 (2)	C6—C5	1.384 (2)
N4—N3	1.3681 (18)	C6—H6	0.93
C20—C25	1.390 (2)	C5—C4	1.371 (3)
C20—C21	1.393 (2)	C5—H5	0.93
C20—N3	1.406 (2)	C24—C23	1.377 (3)
C14—C15	1.389 (2)	C24—H24	0.93
C14—C19	1.394 (2)	C19—C18	1.377 (2)
C14—C13	1.469 (2)	C19—H19	0.93
C33—C34	1.391 (2)	C34—C35	1.374 (2)
C33—C38	1.395 (2)	C34—H34	0.93
C33—C32	1.458 (2)	C2—H2	0.93
N3—C26	1.4406 (19)	C16—C17	1.380 (3)
N1—N2	1.3689 (18)	C16—H16	0.93
N1—C7	1.413 (2)	C36—C35	1.373 (3)
N1—C1	1.441 (2)	C36—H36	0.93
N2—C13	1.279 (2)	C21—C22	1.381 (2)
C32—H32	0.93	C21—H21	0.93
C7—C12	1.379 (2)	C12—C11	1.380 (3)
C7—C8	1.380 (2)	C12—H12	0.93
C13—H13	0.93	C17—C18	1.372 (3)
C1—C6	1.375 (2)	C17—H17	0.93
C1—C2	1.380 (2)	C8—C9	1.379 (3)
C38—C37	1.377 (2)	C8—H8	0.93
C38—H38	0.93	C4—H4	0.93
C25—C24	1.377 (2)	C29—C28	1.369 (2)
C25—H25	0.93	C29—C30	1.379 (3)
C3—C4	1.368 (3)	C29—H29	0.93
C3—C2	1.384 (2)	C28—H28	0.93
C3—H3	0.93	C30—H30	0.93
C31—C26	1.376 (2)	C18—H18	0.93
C31—C30	1.386 (2)	C35—H35	0.93
C31—H31	0.93	C9—C10	1.365 (3)
C27—C26	1.382 (2)	C9—H9	0.93
C27—C28	1.384 (2)	C11—C10	1.364 (3)
C27—H27	0.93	C11—H11	0.93
C15—C16	1.385 (3)	C10—H10	0.93
C15—H15	0.93	C22—C23	1.368 (3)
C37—C36	1.368 (3)	C22—H22	0.93
C37—H37	0.93	C23—H23	0.93
			
C32—N4—N3	119.93 (13)	C14—C19—H19	119.8
C25—C20—C21	118.74 (15)	C35—C34—C33	120.61 (16)
C25—C20—N3	119.73 (14)	C35—C34—H34	119.7
C21—C20—N3	121.53 (14)	C33—C34—H34	119.7
C15—C14—C19	118.52 (16)	C1—C2—C3	119.64 (15)
C15—C14—C13	119.47 (16)	C1—C2—H2	120.2
C19—C14—C13	122.00 (14)	C3—C2—H2	120.2
C34—C33—C38	117.84 (14)	C17—C16—C15	120.51 (18)
C34—C33—C32	122.66 (14)	C17—C16—H16	119.7
C38—C33—C32	119.48 (14)	C15—C16—H16	119.7
N4—N3—C20	117.82 (12)	C37—C36—C35	119.54 (17)
N4—N3—C26	120.99 (12)	C37—C36—H36	120.2
C20—N3—C26	120.60 (12)	C35—C36—H36	120.2
N2—N1—C7	116.32 (13)	C22—C21—C20	119.51 (16)
N2—N1—C1	122.62 (12)	C22—C21—H21	120.2
C7—N1—C1	120.01 (13)	C20—C21—H21	120.2
C13—N2—N1	120.28 (14)	C31—C26—C27	120.16 (14)
N4—C32—C33	121.20 (14)	C31—C26—N3	120.32 (14)
N4—C32—H32	119.4	C27—C26—N3	119.52 (14)
C33—C32—H32	119.4	C7—C12—C11	120.01 (17)
C12—C7—C8	118.27 (15)	C7—C12—H12	120
C12—C7—N1	121.85 (15)	C11—C12—H12	120
C8—C7—N1	119.87 (15)	C18—C17—C16	119.36 (18)
N2—C13—C14	119.90 (15)	C18—C17—H17	120.3
N2—C13—H13	120.1	C16—C17—H17	120.3
C14—C13—H13	120.1	C9—C8—C7	120.65 (18)
C6—C1—C2	119.68 (15)	C9—C8—H8	119.7
C6—C1—N1	119.74 (14)	C7—C8—H8	119.7
C2—C1—N1	120.58 (14)	C3—C4—C5	119.94 (16)
C37—C38—C33	120.81 (16)	C3—C4—H4	120
C37—C38—H38	119.6	C5—C4—H4	120
C33—C38—H38	119.6	C28—C29—C30	119.89 (16)
C24—C25—C20	120.52 (16)	C28—C29—H29	120.1
C24—C25—H25	119.7	C30—C29—H29	120.1
C20—C25—H25	119.7	C29—C28—C27	120.42 (16)
C4—C3—C2	120.54 (16)	C29—C28—H28	119.8
C4—C3—H3	119.7	C27—C28—H28	119.8
C2—C3—H3	119.7	C29—C30—C31	120.15 (16)
C26—C31—C30	119.71 (16)	C29—C30—H30	119.9
C26—C31—H31	120.1	C31—C30—H30	119.9
C30—C31—H31	120.1	C17—C18—C19	120.8 (2)
C26—C27—C28	119.67 (15)	C17—C18—H18	119.6
C26—C27—H27	120.2	C19—C18—H18	119.6
C28—C27—H27	120.2	C36—C35—C34	120.75 (18)
C16—C15—C14	120.34 (19)	C36—C35—H35	119.6
C16—C15—H15	119.8	C34—C35—H35	119.6
C14—C15—H15	119.8	C10—C9—C8	120.96 (19)
C36—C37—C38	120.43 (16)	C10—C9—H9	119.5
C36—C37—H37	119.8	C8—C9—H9	119.5
C38—C37—H37	119.8	C10—C11—C12	121.58 (19)
C1—C6—C5	120.22 (16)	C10—C11—H11	119.2
C1—C6—H6	119.9	C12—C11—H11	119.2
C5—C6—H6	119.9	C11—C10—C9	118.46 (17)
C4—C5—C6	119.97 (16)	C11—C10—H10	120.8
C4—C5—H5	120	C9—C10—H10	120.8
C6—C5—H5	120	C23—C22—C21	121.57 (18)
C23—C24—C25	120.58 (17)	C23—C22—H22	119.2
C23—C24—H24	119.7	C21—C22—H22	119.2
C25—C24—H24	119.7	C22—C23—C24	119.07 (17)
C18—C19—C14	120.44 (17)	C22—C23—H23	120.5
C18—C19—H19	119.8	C24—C23—H23	120.5
			
C32—N4—N3—C20	−170.05 (14)	C6—C1—C2—C3	1.7 (2)
C32—N4—N3—C26	1.2 (2)	N1—C1—C2—C3	−177.69 (14)
C25—C20—N3—N4	−177.82 (13)	C4—C3—C2—C1	−0.8 (3)
C21—C20—N3—N4	2.3 (2)	C14—C15—C16—C17	0.3 (3)
C25—C20—N3—C26	10.9 (2)	C38—C37—C36—C35	−1.1 (3)
C21—C20—N3—C26	−168.98 (15)	C25—C20—C21—C22	0.0 (3)
C7—N1—N2—C13	−172.96 (14)	N3—C20—C21—C22	179.94 (17)
C1—N1—N2—C13	−4.7 (2)	C30—C31—C26—C27	0.3 (2)
N3—N4—C32—C33	−177.21 (13)	C30—C31—C26—N3	179.36 (15)
C34—C33—C32—N4	5.7 (2)	C28—C27—C26—C31	0.0 (2)
C38—C33—C32—N4	−176.26 (14)	C28—C27—C26—N3	−179.11 (14)
N2—N1—C7—C12	4.5 (2)	N4—N3—C26—C31	−87.86 (19)
C1—N1—C7—C12	−164.03 (15)	C20—N3—C26—C31	83.1 (2)
N2—N1—C7—C8	−176.26 (15)	N4—N3—C26—C27	91.25 (19)
C1—N1—C7—C8	15.2 (2)	C20—N3—C26—C27	−97.77 (18)
N1—N2—C13—C14	−178.09 (13)	C8—C7—C12—C11	−2.4 (3)
C15—C14—C13—N2	−173.94 (15)	N1—C7—C12—C11	176.78 (17)
C19—C14—C13—N2	5.9 (2)	C15—C16—C17—C18	0.2 (3)
N2—N1—C1—C6	88.2 (2)	C12—C7—C8—C9	3.1 (3)
C7—N1—C1—C6	−103.97 (18)	N1—C7—C8—C9	−176.16 (17)
N2—N1—C1—C2	−92.37 (19)	C2—C3—C4—C5	−0.3 (3)
C7—N1—C1—C2	75.4 (2)	C6—C5—C4—C3	0.5 (3)
C34—C33—C38—C37	−0.5 (2)	C30—C29—C28—C27	−0.3 (3)
C32—C33—C38—C37	−178.62 (14)	C26—C27—C28—C29	0.0 (3)
C21—C20—C25—C24	−0.1 (2)	C28—C29—C30—C31	0.6 (3)
N3—C20—C25—C24	180.00 (14)	C26—C31—C30—C29	−0.6 (3)
C19—C14—C15—C16	−0.6 (2)	C16—C17—C18—C19	−0.4 (3)
C13—C14—C15—C16	179.23 (15)	C14—C19—C18—C17	0.0 (3)
C33—C38—C37—C36	1.5 (3)	C37—C36—C35—C34	−0.4 (3)
C2—C1—C6—C5	−1.5 (3)	C33—C34—C35—C36	1.5 (3)
N1—C1—C6—C5	177.89 (15)	C7—C8—C9—C10	−1.8 (3)
C1—C6—C5—C4	0.4 (3)	C7—C12—C11—C10	0.6 (3)
C20—C25—C24—C23	0.3 (3)	C12—C11—C10—C9	0.7 (3)
C15—C14—C19—C18	0.5 (2)	C8—C9—C10—C11	−0.1 (3)
C13—C14—C19—C18	−179.39 (16)	C20—C21—C22—C23	−0.1 (3)
C38—C33—C34—C35	−1.0 (3)	C21—C22—C23—C24	0.3 (3)
C32—C33—C34—C35	177.06 (16)	C25—C24—C23—C22	−0.4 (3)

### Hydrogen-bond geometry (Å, °)

**Table:**

*Cg*1, *Cg*2 and *Cg*4 are the centroids of the C1–C6, C7–C12 and C20–C25 rings, respectively.

**Table:**

*D*—H···*A*	*D*—H	H···*A*	*D*···*A*	*D*—H···*A*
C15—H15···Cg4^i^	0.93	2.93	3.851 (2)	171
C22—H22···Cg2^ii^	0.93	2.88	3.788 (3)	166
C37—H37···Cg1^iii^	0.93	2.99	3.828 (2)	150

Symmetry codes: (i) −*x*+1, −*y*, −*z*+1; (ii) *x*+1, *y*−1, *z*; (iii) −*x*+1, −*y*+1, −*z*.

## References

[bb1] Allen, F. H., Kennard, O., Watson, D. G., Brammer, L., Orpen, A. G. & Taylor, R. (1987). *J. Chem. Soc. Perkin Trans. 2*, pp. S1–19.

[bb2] Burla, M. C., Caliandro, R., Camalli, M., Carrozzini, B., Cascarano, G. L., De Caro, L., Giacovazzo, C., Polidori, G. & Spagna, R. (2005). *J. Appl. Cryst.* **38**, 381–388.

[bb3] Clulow, A. J., Selby, J. D., Cushion, M. G., Schwarz, A. D. & Mountford, P. (2008). *Inorg. Chem.* **47**, 12049–12062. 10.1021/ic801735c 18998672

[bb4] Farrugia, L. J. (1997). *J. Appl. Cryst.* **30**, 565.

[bb5] Farrugia, L. J. (1999). *J. Appl. Cryst.* **32**, 837–838.

[bb6] Guniz, S. & Rollas, S. (2002). *Farmaco*, **57**, 583–587. 10.1016/s0014-827x(02)01253-3 12164219

[bb7] Ibañez, G. A., Escandar, G. M. & Olivieri, A. C. (2002). *J. Mol. Struct.* **605**, 17–26.

[bb8] Mendoza, A., Cabrera-Vivas, B. M., Meléndrez-Luevano, R., Ramírez, J. C. & Flores-Alamo, M. (2011). *Acta Cryst.* E**67**, o1287. 10.1107/S1600536811015352 PMC3120608 21754694

[bb9] North, A. C. T., Phillips, D. C. & Mathews, F. S. (1968). *Acta Cryst.* A**24**, 351–359.

[bb10] Rollas, S., Gulerman, N. & Erdeniz, H. (2002). *Farmaco*, **57**, 171–174. 10.1016/s0014-827x(01)01192-2 11902660

[bb11] Sheldrick, G. M. (2008). *Acta Cryst.* A**64**, 112–122. 10.1107/S0108767307043930 18156677

[bb12] Siemens (1994). *XSCANS* Siemens Analytical X-ray Instruments Inc., Madison, Wisconsin, USA.

[bb13] Vicini, P., Zani, F., Cozzini, P. & Doytchinova, I. (2002). *Eur. J. Med. Chem.* **37**, 553–564. 10.1016/s0223-5234(02)01378-8 12126774

